# An amodal shared resource model of language-mediated visual attention

**DOI:** 10.3389/fpsyg.2013.00528

**Published:** 2013-08-16

**Authors:** Alastair C. Smith, Padraic Monaghan, Falk Huettig

**Affiliations:** ^1^Max Planck Institute for PsycholinguisticsNijmegen, Netherlands; ^2^International Max Planck Research School for Language SciencesNijmegen, Netherlands; ^3^Department of Psychology, Lancaster UniversityLancaster, UK; ^4^Donders Institute for Brain, Cognition, and Behaviour, Radboud UniversityNijmegen, Netherlands

**Keywords:** language, vision, computational modeling, attention, eye movements, semantics

## Abstract

Language-mediated visual attention describes the interaction of two fundamental components of the human cognitive system, language and vision. Within this paper we present an amodal shared resource model of language-mediated visual attention that offers a description of the information and processes involved in this complex multimodal behavior and a potential explanation for how this ability is acquired. We demonstrate that the model is not only sufficient to account for the experimental effects of Visual World Paradigm studies but also that these effects are emergent properties of the architecture of the model itself, rather than requiring separate information processing channels or modular processing systems. The model provides an explicit description of the connection between the modality-specific input from language and vision and the distribution of eye gaze in language-mediated visual attention. The paper concludes by discussing future applications for the model, specifically its potential for investigating the factors driving observed individual differences in language-mediated eye gaze.

## Integrative processing in a model of language-mediated visual attention

### Language-mediated visual attention

Within daily communicative interactions a vast array of information sources have to be integrated in order to understand language and relate it to the world around the interlocutors. Such multimodal interactions within the speaker and listener have been shown to be vital for language development (Markman, 1994; Bloom, 2000; Monaghan and Mattock, 2012; Mani et al., 2013) as well as for adult sentence and discourse processing (Anderson et al., 2011; Huettig et al., 2011b; Lupyan, 2012). Eye gaze has been used to demonstrate the nature of the processes supporting online integration of linguistic and visual information (Halberda, 2006; Huettig et al., 2011a). Such observations of eye gaze have opened up the possibility to investigate how multiple sources of information, within the environment and within the language signal, interact in the human cognitive system. We begin by describing the observed properties of eye gaze behavior that have informed our understanding of the representations and processes involved in language—vision interactions. We then present a computational model of language-mediated visual attention that implements the representations and processes identified within a parsimonious neural network architecture. Finally, we demonstrate that many of the characteristic features of language-mediated eye gaze can be captured by the emergent properties of this parsimonious architecture and therefore do not necessitate separate information processing channels or modular processing systems.

One influential paradigm for measuring language and vision interactions is the Visual World Paradigm (VWP; Cooper, 1974; Tanenhaus et al., 1995), in which participants are presented with a visual display comprising a set of objects and/or actors whilst hearing an auditory stimulus and during this period their eye gaze is recorded. Although eye gaze is a measure of overt attention and thus not a direct reflection of linguistic processing, the VWP has been utilized largely to investigate questions that explore how the cognitive system processes spoken language (see Huettig et al., 2011b, for review). A few studies, however, have investigated multimodal interactions. Such studies tend to focus on how eye gaze alters as the auditory stimulus unfolds and how varying the relationships between objects in the display can highlight which modalities of information are implicated at varying time points in language processing.

Many visual world studies have demonstrated that eye gaze can be modulated by phonological relationships between items presented in the visual display and spoken target words. Allopenna et al. (1998), for instance, showed that when hearing a target word (e.g., “beaker”) participants looked more toward items in the display whose names overlapped phonologically with the target word either in initial (e.g., beetle) or final (e.g., speaker) positions, than items that were not related phonologically (e.g., carriage) to the spoken target word. They found that, relative to unrelated items, there was increased fixation of phonological competitors. Furthermore, fixations to onset competitors occurred earlier than those to rhyme competitors and the probability of fixating onset competitors was greater than the probability of fixating rhyme competitors.

Visual relationships between items have also been shown to influence fixation behavior (Dahan and Tanenhaus, 2005; Huettig and Altmann, 2007). Dahan and Tanenhaus (2005) presented scenes containing a target (e.g., a snake), a visual competitor (e.g., a rope) and two unrelated distractors (e.g., a couch and an umbrella), while Huettig and Altmann (2007) presented scenes without a visual depiction of the target but with a visual competitor and three unrelated distractors. Thus, items within the display that shared visual features associated with the spoken target word, yet whose names did not overlap phonologically with the target word, attracted greater fixation than unrelated items.

**Figure 1:**
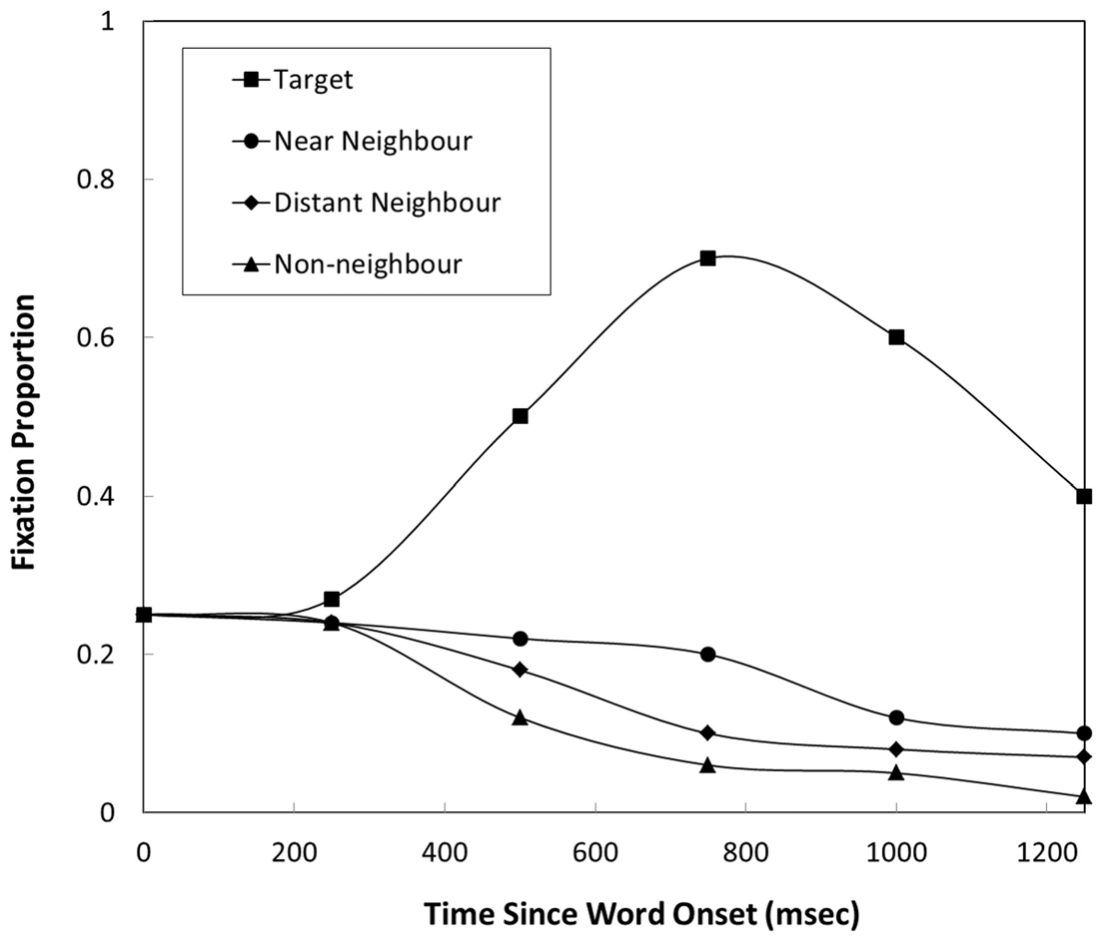
**Figure adapted from Mirman and Magnuson (2009).** Figure displays approximate fixation proportions for targets, near semantic neighbors, distant semantic neighbors and unrelated items displayed by participants in Mirman and Magnuson (2009).

Another dimension in which relationships between visually displayed items and spoken target words has been shown to modulate eye gaze is that of semantics. Huettig and Altmann (2005) and Yee and Sedivy (2006) demonstrated that items that share semantic (but not visual or phonological) relationships with target words are fixated more than unrelated items. Yee and Sedivy (2006) presented displays containing a target item (e.g., lock), a semantically related item (e.g., key) and two unrelated distractors. Similarly, Huettig and Altmann (2005) presented scenes containing both a target (e.g., piano) and a semantic competitor (e.g., trumpet) or scenes containing only a semantic competitor (e.g., only the trumpet) and unrelated items. In both target present and target absent conditions increased fixations of semantically related items were observed. *Post-hoc* analyses revealed that the likelihood of fixation was proportional to the degree of semantic overlap as measured by feature production norms (cf. Cree and McRae, 2003) and corpus-based measures of word semantics (Huettig et al., 2006). Further evidence for a relationship between semantic overlap and eye gaze is provided by Mirman and Magnuson (2009) who directly tested the gradedness of semantic overlap. They presented scenes containing a target item (e.g., bus) paired with either a near semantic neighbor (e.g., van) or a distant semantic neighbor (e.g., bike) and two unrelated items (e.g., ball). The likelihood of fixating each item was predicted by the level of semantic overlap, with near semantic neighbors fixated with greater probability than far semantic neighbors, while both were fixated with lower probability than targets and greater probability than distractors (see Figure 1).

In order to probe the relationships between previously observed phonological, visual and semantic word level effects in the VWP, Huettig and McQueen (2007) presented scenes containing phonological onset, semantic and visual competitors in addition to an unrelated distractor. They observed distinct patterns of fixation for each competitor type, with participants initially looking more toward phonological onset competitors before later displaying greater fixation of visual and semantic competitors. From these results they concluded that language-mediated visual attention is determined by matches between information extracted from the visual display and speech signal at phonological, visual and semantic levels of processing.

Taken together, this significant body of evidence shows that visual, semantic and phonological information is co-activated and integrated during spoken word processing. However, the nature of the information and mechanisms involved in visual world and language processing interactions are as yet underspecified (Huettig et al., 2011a, 2012). How is information activated within one modality integrated with information activated within another, what form does this information take, how does such information interact and how is this interaction connected to eye gaze behavior? There are two principle possibilities for interactions to occur: They may be a consequence of modality specific systems interacting via direct connections; alternatively, interactions may occur as a consequence of amodal shared resources facilitating interaction between the various information modalities (Lambon Ralph and Patterson, 2008; Plaut, 2002). Computational implementation of theoretical models offers a means of testing their plausibility and often provides a means of probing aspects of theoretical models that may lie beyond the reach of behavioral studies. The VWP provides a high degree of experimental control that offers a well constrained environment in which models can operate. Models of the processes involved in performing VWP tasks force researchers to define explicitly how information carried in the visual and auditory stimuli is connected to distributions of eye gaze.

In this paper, we first present previous modeling approaches that have accounted for the various VWP results presented above before elaborating the modular vs. shared-resource computational approaches to multimodal information processing. We then present our model of the shared resource account of language-mediated visual attention and demonstrate that it is not only sufficient to account for the experimental effects of VWP studies but also that these effects are emergent properties of the architecture of the model itself.

### Previous models of language-mediated visual attention

Most previous models of the VWP have focused on explaining interactions between vision and a single feature of language processing. For instance, Allopenna et al. (1998) chose TRACE (McClelland and Elman, 1986) to simulate the mechanisms driving differences in the effect of phonological onset and rhyme overlap. TRACE is a continuous mapping model of speech perception, implemented in an interactive activation network that hierarchically processes speech at the level of phonemic features, phonemes and words. The model successfully replicated the contrasting patterns of fixation displayed by participants toward onset and rhyme competitors and offered explanation for contrasts between the location of overlap and its influence on eye gaze. However, the model focuses purely on phonological processing and therefore as a model of language-mediated visual attention it provides no description of the role other information sources play in this process.

Magnuson et al. (2003) further examined the mechanisms underlying observed cohort and rhyme effects. They demonstrated that differences in sensitivity to both cohort and rhyme competitors displayed by adults over the course of word learning could be captured in the emergent behavior displayed by an SRN (Elman, 1990) trained to map between phonetic features and localist word level representations. Unlike TRACE, in which connection weights were fixed by the modeler, connection weights within the SRN were adjusted using an error based learning algorithm. This not only reduces the number of parameters directly manipulated by the modeler and therefore the number of assumptions underpinning the model but also allowed authors to chart model behavior over the course of word learning. Using this approach they were able to demonstrate that a fundamental difference between adult and child lexical representations was not required to explain differences in sensitivity to rhyme and cohort competitors. Instead such differences were captured by their model due to the strengthening of lexical representations over the course of word learning. Again, however, the focus of this work is on aspects of phonological processing in the VWP. Therefore, as a model of language-mediated visual attention it ignores the role of other knowledge types in this process.

Similarly, Mirman and Magnuson (2009) used the attractor network of Cree et al. (1999) to simulate the graded effect of semantic competitors influencing eye gaze. The network consisted of a word form input layer and semantic feature output layer. The model was trained to map 541 words onto their corresponding semantic features derived from feature norming studies. However, as in the case of Magnuson et al. (2003) and TRACE, such models offer representation of items from only a single information source (phonological or semantic similarity) and therefore are unable to account for the full range of intermodal effects demonstrated in the VWP. Also, none of these models offer a description of how information activated by distinct visual and auditory sources can be combined to influence fixation behavior. They therefore do not provide a comprehensive model of the word level effects observed in the VWP.

There have, however, been some notable models of multimodal processing in VWP (Spivey, 2008; Mayberry et al., 2009; Kukona and Tabor, 2011). Spivey (2008) extended TRACE to incorporate visual processing, by connecting lexical activations in TRACE to a normalized recurrent localist attractor network that represented the presence or absence of items within the visual environment. However, in using localist visual representations the model lacks depth of representation in the visual modality to capture subtle relationships between items known to influence fixation behavior in VWP, such as visual similarity effects.

Mayberry et al. (2009) also provided a model of the interaction between visual and linguistic information in the VWP. Their connectionist model (CIANET) displays emergent properties that capture sentence level effects such as case role interpretation. A potential weakness of the model is its use of the same form of representation to encode both visual and linguistic information, thereby masking potential distinct effects of visual vs. linguistic similarities. A further weakness of both CIANET and Spivey (2008) is that neither provide representation at the word level in a semantic dimension, although we know from previous VWP studies that items can differ in both visual and phonological dimensions yet still share semantic properties that influence eye gaze behavior.

Finally, Kukona and Tabor (2011) presents a dynamical systems model of eye gaze in VWP in which localist representations at phonological, lexical-semantic, cross-word and action-space layers interact in a hierarchically structured network. Visual information is modeled by the presence or absence of its corresponding representations within the network. By representing items at this level of abstraction their model is unable to capture complex relationships between representations in the same modality. It seems then that none of the current multimodal models that have been used to explicitly model VWP data offer sufficient depth of representation in the multiple modalities involved to capture the subtle relationships between items shown to influence eye gaze at the word level in VWP.

Yet, previous models and their success in replicating individual VWP data sets have provided valuable insight into the type of architecture capable of supporting language-mediated visual attention. The architecture must allow for competition at multiple levels of representation (Allopenna et al., 1998), allow both excitatory and inhibitory connections (Mirman and Magnuson, 2009), facilitate parallel activation of representations (Kukona and Tabor, 2011) and integrate information from multiple sources (Mayberry et al., 2009). Such integration could be accomplished by connectivity between individual representational modalities, or via processing interconnectivity through a shared resource.

### Modular vs. shared-resource models

A framework able to capture the architectural features of language-mediated visual attention identified in the previous section is the Hub-and-spoke (H&S) framework. H&S models are defined by an amodal central resource (hub) that integrates and translates information between multiple modality specific sources (spokes). The framework arose as one side of a debate regarding the neural structures that support human conceptual and semantic knowledge. Lambon Ralph and Patterson (2008) compared two alternative theoretical models to account for visual and linguistic semantic processing in unimpaired and patient populations. One consisted purely of modality specific processing regions connected via direct connections, the second instead connected regions via a modality invariant central hub, the H&S model. The authors argue that although a web of direct connections may provide a simpler architectural solution, only a model that contains a central connecting hub offers a system capable of performing the multilevel non-linear computations required for semantic generalization and inference based on conceptual structure rather than surface similarities. There is also converging empirical evidence for both the existence of a semantic hub and its implementation in specific neural populations in the anterior temporal lobe (ATL). This evidence includes neuropsychological studies of patients suffering from semantic dementia (SD) (Lambon Ralph et al., 2010) who possess lesions in the ATL and display deficits in performance on tasks requiring semantic generalization. Similarly, non-patient groups that experience artificial lesions in the ATL using rTMS (Pobric et al., 2007) have reported similar deficits in performance on such tasks. Finally, neuroimaging studies (Vandenberghe et al., 1996), have observed activity in the ATL on tasks that require semantic generalization. These data support the notion that a central resource that integrates modality specific information is a crucial component of the architecture supporting semantic processing.

Models that postulate integrative processing from multiple sources are embedded in a broader literature that has debated the inherence of sensory and motor systems to conceptual representations. Studies of “embodied cognition,” for instance, have made the case for the importance of motor and sensory systems for cognitive processing (e.g., Barsalou et al., 2003, but see Mahon and Caramazza, 2008). An important debate concerns the format of mental representations with some proponents of the embodied cognition hypothesis suggesting that conceptual knowledge consists entirely of “representational codes that are specific to our perceptual systems” (Prinz, 2002, p. 119). This contrasts with representational theories which assume that sensory and motor knowledge is amodal and abstracted away from modality-specific systems (e.g., Kintsch, 2008). A third view posits the existence of both amodal and modal representations providing an explanation of how we are able to acquire knowledge which goes beyond sensory and motor experience (Goldstone and Barsalou, 1998; Dove, 2009). This view is supported by recent demonstrations that co-activation of multimodal systems can be effectively simulated by models with an amodal shared resource (Yoon et al., 2002; Monaghan and Nazir, 2009). Given that activation in a spoke of a H&S model represents modality specific processing of an item, and activation within the hub captures an items amodal properties, then the interaction of modal (spoke) and amodal (hub) representations is a natural consequence of the architecture of H&S models. A recent review of the mechanisms and representations involved in language-mediated visual attention (Huettig et al., 2012) concluded that the most promising theoretical model to date postulates that language-mediated visual attention is dependent on a system in which both linguistic, non-linguistic and attentional information are all instantiated within the same coding substrate, which is required in order for information to be bound across modalities. The H&S framework offers a parsimonious solution by connecting modalities through a central processing hub.

Research examining the plausibility of alternative theoretical models of multimodal cognition has profited from testing their predictions using explicit neural network implementations of the H&S framework. In the following sections we detail the nature of these studies and how they have contributed to our understanding of the mechanisms that support semantic processing. We also identify the features of the Hub and Spoke framework that make it a valuable tool for modeling various aspects of multimodal cognition. We then test the framework's scope by using it as a foundation for a model of language-mediated visual attention.

### Insights from hub and spoke models

The H&S framework offers a parsimonious architecture in which single modality models can be drawn together to examine the consequences of multimodal interaction. Producing an explicit model of the mechanisms thought to underlie a given process allows one to test theoretical positions and probe deeper the mechanisms that may be involved in a controlled and tractable manner.

The framework provides a single system architecture with only minimal initial assumptions on connectivity. As the systems architecture imposes minimal constraints on the flow of information within the network, emergent behavior is largely driven by (1) representational structure and/or (2) the tasks or mappings performed by the system during the learning process. Therefore, within the framework the scope of such factors in driving emergent properties of complex multimodal systems can be examined largely independent of modality specific architectural constraints.

Two alternative means of exploring the role of representational structure are presented in previous H&S models. Plaut (2002) simulates impairments displayed by optic aphasics in an H&S model that mapped between distinct vision, action (gesturing), touch and phonological (naming) layers. The author takes a fundamentalist approach (see Kello and Plaut, 2000) ensuring he has total control over any relationships embedded in representations within or across modalities. This allows the study to isolate emergent properties driven by individual aspects of representational structure. In Plaut (2002) the variable manipulated was systematicity in representation between modalities. He embedded systematic mappings between tactile, vision and action representations while those between phonology and other modalities were arbitrary. This feature of representations allowed the model to capture key features of patient behavior with the lack of systematicity in phonological representations leading to poor performance on naming tasks post lesioning.

In contrast, Rogers et al. (2004) (approach replicated in Dilkina et al., 2008, 2010) employs a realist approach with representations derived from feature norming studies. Within the study deficits in semantic processing displayed by SD patients are modeled using an H&S framework. The model consisted of a visual layer connected via a central resource to a verbal descriptor layer comprising names, perceptual, functional, and encyclopaedic information about objects. Although a realist approach requires the modeler to relinquish control over the structure embedded within the corpus, the resulting structure aims to provide a closer representation of that available within the natural learning environment. Consequently, this reduces the extent to which emergent properties are determined by prior assumptions of the modeler and provides a means of examining the content of behavior determined by naturally occurring structure within the environment. The model presented in Rogers et al. (2004), generates the counterintuitive prediction that damaged semantic systems are more likely to perform better at specific relative to general sorting in the case of fruits. This subtle aspect of behavior is captured as a result of the model implementing rich representations of the structure of information available within the environment.

With small corpora it is also possible to analyse the structure embedded within representations derived from natural data to identify features that may have an influence on emergent behavior. This is demonstrated in Dilkina et al. (2010), in which individual differences displayed by SD patients were modeled in an H&S framework that mapped between orthographic, action, vision and phonological layers. The behavior of a subset of SD patients whose performance on lexical and semantic tasks did not correlate by item had been argued to result from two functionally distinct systems (e.g., Coltheart, 2004). The study demonstrated the compatibility of a single system model with the empirical data and offered an alternative explanation based on the role of spelling and concept consistency. The authors argued that observed effects emerged due to the structure embedded within representations rather than modality specific architectural constraints.

Behavior is not only constrained by representational structure but also by the manner in which the system interacts with representations, for example the form and quantity of mappings demanded by the learning environment. H&S models have demonstrated how the framework is able to examine the consequences of such environmental factors. Dilkina et al. (2010) captures contrasts in mappings over the course of development. Training is split into two stages, with mapping from orthography to phonology only performed in the second stage. This aims to reflect the fact that learning to read only occurs at a later stage of development. The proportion and period in which certain mappings such as vision to action occur may remain relatively constant both over the course of development and populations. However, it is also true that in many cases there will be variation in the form and quantity of mapping between individuals and more broadly populations. Dilkina et al. (2008) uses this feature of the learning environment to explore one possible factor driving individual difference in SD, that being the level of prior reading experience. Within the study, prior reading experience is modeled by manipulating the amount of training on orthographic to phonological mapping. Demonstrating the influence of such factors, manipulation of this variable was able to account for four of the five SD patients behavior. Clearly, such variation in the type of mapping performed and stage at which it's performed can have dramatic consequences for emergent properties of the system. However, predicting the nature of such properties in complex multimodal systems is far from trivial. H&S offers a means of examining the consequences of variation in such environmental variables.

To conclude, behavioral data from the VWP suggests that language-mediated visual attention is driven by the interaction of information extracted from the visual environment and speech signal at semantic, visual and phonological levels of processing. The H&S framework provides a parsimonious architecture within which the emergent properties of this complex interaction can be modeled. Previous modeling of the VWP has identified further properties of the architecture involved. These include allowing competition at multiple levels of representation, parallel activation of representations, the integration of information from multiple sources and allowing inhibitory and excitatory associations. A neural network architecture such as those used in previous implementations of the H&S framework naturally captures these characteristics.

### Investigation goals

We next present a computational model of the various sources of information contributing to eye gaze in the VWP. Our aims were as follows. First, we tested whether a H&S model, with minimal computational architectural assumptions, was sufficient for replicating the effects of phonological and semantic influences on language processing in the VWP, or whether individual models combining the modal-specific features of the models of Allopenna et al. (1998) and Mirman and Magnuson (2009) would be required to effectively simulate the range of effects across these distinct modalities. Second, we tested whether the model could further generalize to simulate effects of visual information similarity in the VWP (Dahan and Tanenhaus, 2005; Huettig and Altmann, 2007). Third, we tested whether the model was further able to replicate sensitivity to the effects of presenting or not presenting the object corresponding to the target word in the various VWP experimental manipulations of visual, phonological, and semantic competitors. In each case, the model's performance is a consequence of the integrated processing of multimodal information, resulting from specified properties of the representations themselves and also the computational properties of the mappings between them.

The model we present connects visual, semantic and linguistic information to drive eye gaze behavior. Specifically, the model was tested on its ability to replicate the following features of language-mediated visual attention demonstrated in Visual World studies: (1) Fixation of onset and rhyme competitors above unrelated distractor levels in target present scenes (Allopenna et al., 1998); (2) Fixation of visual competitors above unrelated distractor levels in both target present (Dahan and Tanenhaus, 2005) and target absent (Huettig and Altmann, 2007) scenes; and (3) Fixation of semantic competitors above unrelated distractor levels and relative to semantic relatedness in both target present (Yee and Sedivy, 2006; Mirman and Magnuson, 2009) and absent (Huettig and Altmann, 2005) scenes. We present two simulations—one with no environmental noise, and one with background environmental noise. We later show that environmental noise is necessary for replicating all aspects of behavioral data.

### Modeling language-mediated visual attention in a noiseless learning environment

#### Method

**Figure 2:**
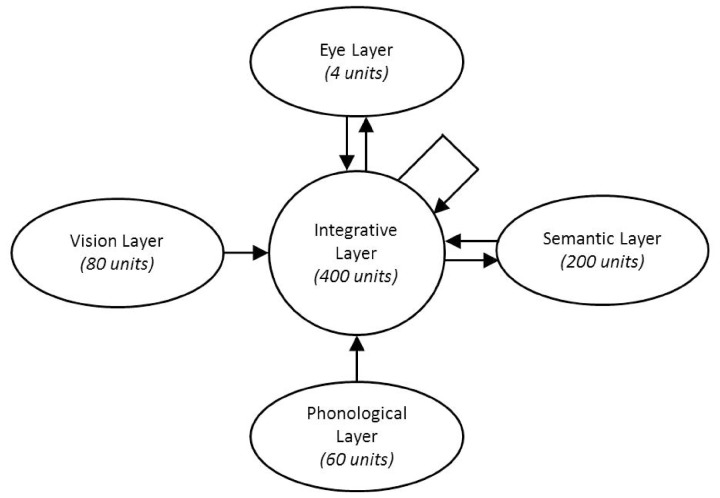
**Network Architecture**.

***Architecture.*** The architecture of the H&S neural network used within this study is displayed in Figure 2. Akin to previous H&S models it was composed of a central resource (integrative layer) consisting of 400 units that integrated modality specific information from four “visible” layers, which encoded input and output representational information. The vision layer consisted of 80 units and modeled the extraction of visual information from four spatial locations within the environment. It contained four slots each containing 20 units which extracted visual information from each of four distinct locations in the visual field. The phonological layer consisted of 60 units and encoded phonological information from the speech signal. This layer comprised six phoneme slots each represented by 10 units, such that words up to 6 phonemes in length could be represented unfolding across time. A semantic layer of 200 units represented semantic information of items, with units representing semantic features of the concept. The eye layer consisted of four units. Each unit within the eye layer was associated with one of the four locations within the model's visual field. The level of activation of an eye unit represented the probability of fixating the spatial location with which the unit was associated. All visible layers were fully connected to the central integrative layer, and the central integrative layer was in turn fully self-connected and fully connected to the eye and semantic layers.

At each time step of the model's processing, activation passed between all layers of units in the model. During training, there were 14 time steps to enable activation to cycle between representations in the model. During testing, the number of time steps was extended to enable insight into the time-course of representational information interacting between the modalities within the model.

***Artificial corpus.*** A fundamentalist approach (Kello and Plaut, 2000) was taken in construction of representations to ensure all aspects of the representations were controlled within simulations. Therefore, an artificial corpus composed of 200 items each with unique phonological, visual and semantic representations was constructed and used to train and test the model. Visual representations were generated to represent visual features in different spatial locations, with features representing both coarse (low frequency) and fine (high frequency) visual features. Phonological representations were encoded to create time-dependent slots for the unfolding speech, with categorical representations of phonemes shared across different words. Semantics in the model were rich, in that they were distributed feature based representations with structured relationships between items. They were also relatively sparse and discrete, reflecting behavioral studies of semantic feature-based representations (Harm and Seidenberg, 2004).

Visual representations were encoded as 20 unit binary feature vectors, with each unit representing the presence or absence of a given visual feature. Features were assigned to items randomly with *p*(active) = 0.5. Phonological representations consisted of a fixed sequence of six phonemes. Words were constructed by randomly sampling phonemes from a phoneme inventory containing a total of 20 possible phonemes. Each phoneme was encoded by a 10 unit binary feature vector, with *p*(active) = 0.5. For semantic representations, a unique subset of 8 semantic features was randomly assigned to each item from the set of 200 possible features.

The level of overlap between items in semantic, visual and phonological dimensions was controlled (see Table 1). Within the corpus were embedded 20 target items each with either visual, near semantic, far semantic, phonological onset or rhyme competitors. Competitors were defined by the increased number of features shared with their assigned target in either a semantic, visual or phonological dimension. A consistent level of representational overlap was implemented across all modalities (other than in the case of far semantic competitors) by ensuring that the distance in terms of shared features between a target and a competitor was on average half the distance of that between a target and unrelated item in the modality that defined the competitor type. Six randomly generated corpora were generated using different initial random seeds, to ensure that no accidental correspondences between particular representations occurred systematically.

**Table 1:** **Controls used in the construction of artificial corpora and mean cosine distance calculated between targets, competitors and unrelated items all six randomly generated corpora used to train and test models**.

**Modality**	**Item**	**Artificial corpus**	
		**Constraint (*Features shared with target*)**	**Cosine distance ($\bar{x}$, σ)**
Phonological	Onset competitor	First 3 phonemes	0.259 (0.026)
	Rhyme competitor	Final 3 phonemes	0.260 (0.028)
	Unrelated	Max. 2 consecutive phonemes	0.496 (0.052)
Semantic	Near neighbor	4 of 8 functional properties	0.500 (0)
	Far neighbor	2 of 8 functional properties	0.750 (0)
	Unrelated	Max. 1 functional property	0.959 (0.072)
Visual	Competitor	Min. 10 of 20 visual features	0.264 (0.040)
	Unrelated	Features shared with *p* = (0.5)	0.506 (0.068)

Onset competitors shared the initial three phonemes with their corresponding target word. No two words shared their initial four phonemes. Rhyme competitors shared the final three phonemes with their assigned target. No two words shared their final four phonemes. No item within the corpus contained more than two identical phonemes per word and no more than two consecutive phonemes overlapped between two unrelated items. These constraints resulted in a cosine distance between phonological representations of 0.259 between onset competitors and targets, 0.260 between rhyme competitors and targets and 0.496 between unrelated items and targets.

The length of vectors used to encode representations in both semantic and visual dimensions was determined by the constraints placed on relationships between items in these modalities. In the case of visual competitors, 10 of 20 visual features were shared between the target and competitor with *p*(shared) = 1, remaining features were shared with *p*(shared) = 0.5. For all visually unrelated items features were shared with *p*(shared) = 0.5. Such controls resulted in a smaller visual feature cosine distance between visual competitors and target items than between unrelated items and targets (see Table 1).

In the semantic dimension, near semantic competitors shared 4 of 8 semantic features with their corresponding target, while 2 of 8 were shared between far semantic competitors and targets. Controls ensured that unrelated items shared a maximum of one semantic feature. Semantic feature cosine distance was least between near neighbors and targets, medial between far neighbors and targets and most between unrelated items and targets (see Table 1).

***Training.*** Model training simulated learning experience in the natural environment that leads to the acquisition of associations between representations across modalities. We assume that individuals acquire semantic, visual and phonological knowledge of a given item through experience of repeated and simultaneous exposure to these multiple forms of representation within the natural learning environment. The model was trained on four cross modal tasks (see Table 2).

**Table 2:** **Temporal organization of events in model training**.

**Task**	**Vision**		**Phonological**		**Semantic**		**Eye**	
	**Description**	**Time step**	**Description**	**Time step**	**Description**	**Time step**	**Description**	**Time step**
Visual to Semantic	4 visual representations randomly selected from corpus, 1 assigned as target	0–14	Random time invariant noise provided as input	0–14	Semantic representation of target provided post display onset	3–14	Location of target activated, all other locations inactive	0–14
Phonological to Semantic	Random time invariant noise provided as input across all 4 input slots	0–14	Speech signal of target provided as a staggered input	0–14	Semantic representation of target provided post disambiguation	5–14	No constraints on activation	
Phonological to Location	4 visual representations randomly selected from corpus, 1 assigned as target	0–14	Speech signal of target provided as a staggered input	0–14	No constraints on activation		Post disambiguation location of target activated, all other locations inactive	5–14
Semantic to Location	4 visual representations randomly selected from corpus, 1 assigned as target	0–14	Random time invariant noise provided as input	0–14	Semantic representation of target provided	0–14	Location of target activated, all other locations inactive post functional onset	2–14

To simulate the events that lead to associations between an item's visual and semantic properties, the model was trained to map from visual to semantic representations using the following procedure. An example of such an event in the natural learning environment may be viewing an item while simultaneously experiencing some aspect of its function (e.g., seeing and eating from a fork). At trial onset the model was presented with four visual representations randomly selected from the corpus assigned to the four spatial locations within the visual field. One of the four items was then randomly selected as a target and the eye unit corresponding to its location fully activated. Throughout the entire test trial small levels of variable noise was provided as input to the phonological layer to simulate ambient background sound. Once sufficient time has allowed for activation to pass from eye and visual layers to the semantic layer (at time step 3) the item's semantic representation was provided as a target.

Models were also trained to map between phonological and semantic representations, simulating the learning that occurs through simultaneous exposure to the sound of a given word and its semantic properties (i.e., hearing and observing the function of “fork”). First, an item was randomly selected as a target from the corpus. The phonological representation of the target was then provided to the phonological input layer as a staggered input, with one additional phoneme provided at each time step. Once activation of the fourth phoneme (uniqueness point for phoneme competitors and corresponding targets) had had sufficient time to influence activation in the semantic layer (time step 5), the item's semantic representation was provided as a target.

Two further tasks trained the model to orientate toward a visual representation of an item in a spatial location according to given phonological or semantic information. As previously stated we assume that in the natural learning environment individuals are repeatedly exposed simultaneously to the visual and phonological or semantic form of an item. Consequently, the learner determines the association between these representations. Mapping from phonology to location was trained by randomly selecting four items from the corpus, randomly assigning them to four locations, and randomly selecting one as the target. The visual representations relating to each of these items was presented as input to the visual layer at trial onset. At the same point in time, input of the phonological representation of the target item was initiated in the phonological layer with one additional phoneme presented per time step. Once activation relating to the fourth phoneme had had time to influence activation in the eye layer (time step 5), the eye unit corresponding to the location of the target was provided as the target.

For mapping from semantics to location, the trial was similar to the phonology to location task, except that all the semantic features were simultaneously activated at time step 1 and time variant noise was presented to the phonological layer for the entire training trial. Once activation from the semantic and visual layer had been provided sufficient time to influence eye layer activation (time step 2), the training signal was provided.

Training tasks were randomly interleaved. Within the natural learning environment we assume that individuals orientate toward or select items based on their semantic features far more frequently than they orientate toward or select items in response to hearing their name. To reflect the assumption that phonologically driven orienting occurs less frequently than semantically driven orienting, training on phonologically driven orienting was four times less likely to occur than all other training tasks.

All connection weights within the network were initially randomized in a uniform distribution [−0.1, 0.1]. Weights were adjusted using recurrent back-propagation with learning rate = 0.05. In order to simulate participants' prior ability to orientate to items based on their phonological and semantic form and identify items' semantic properties based on their visual or phonological form, the models were required to perform with high accuracy on all four of these tasks prior to testing. To obtain this level of performance training was terminated after 1 million trials. In total 6 simulation runs of the model were trained and tested, using each of the six artificial corpora.

#### Results

***Pre-test.*** Following training all models were tested to assess performance on each of the four training tasks for all items within the training corpus. Noise was presented to visual and phonological slots that did not receive target related input. For tasks presenting the target in the visual input, performance was recorded with the target tested once in each of the four locations in the visual field.

For mapping from visual to semantic representations, activation in the semantic layer was closer in terms of cosine similarity to the target item's semantic representation for all items within the training corpus. When tested on mapping from phonological to semantic representations activation in the semantic layer was also most similar to that of the target's semantic representation for all items within the training corpus.

For the phonology to location mapping task, the location of the target was selected on at least 3 of 4 test trials for 99.83% of items in the training corpus. Averaging across all phonology to location test trials the proportion of trials in which the eye unit corresponding to the location of the target was most highly activated was 92%.

For the semantics to location mapping task, the location of the target was selected on at least 3 of 4 test trials for 99.92% of items within the corpus. The overall proportion of successful semantic to location test trials was 89%.

**Figure 3:**
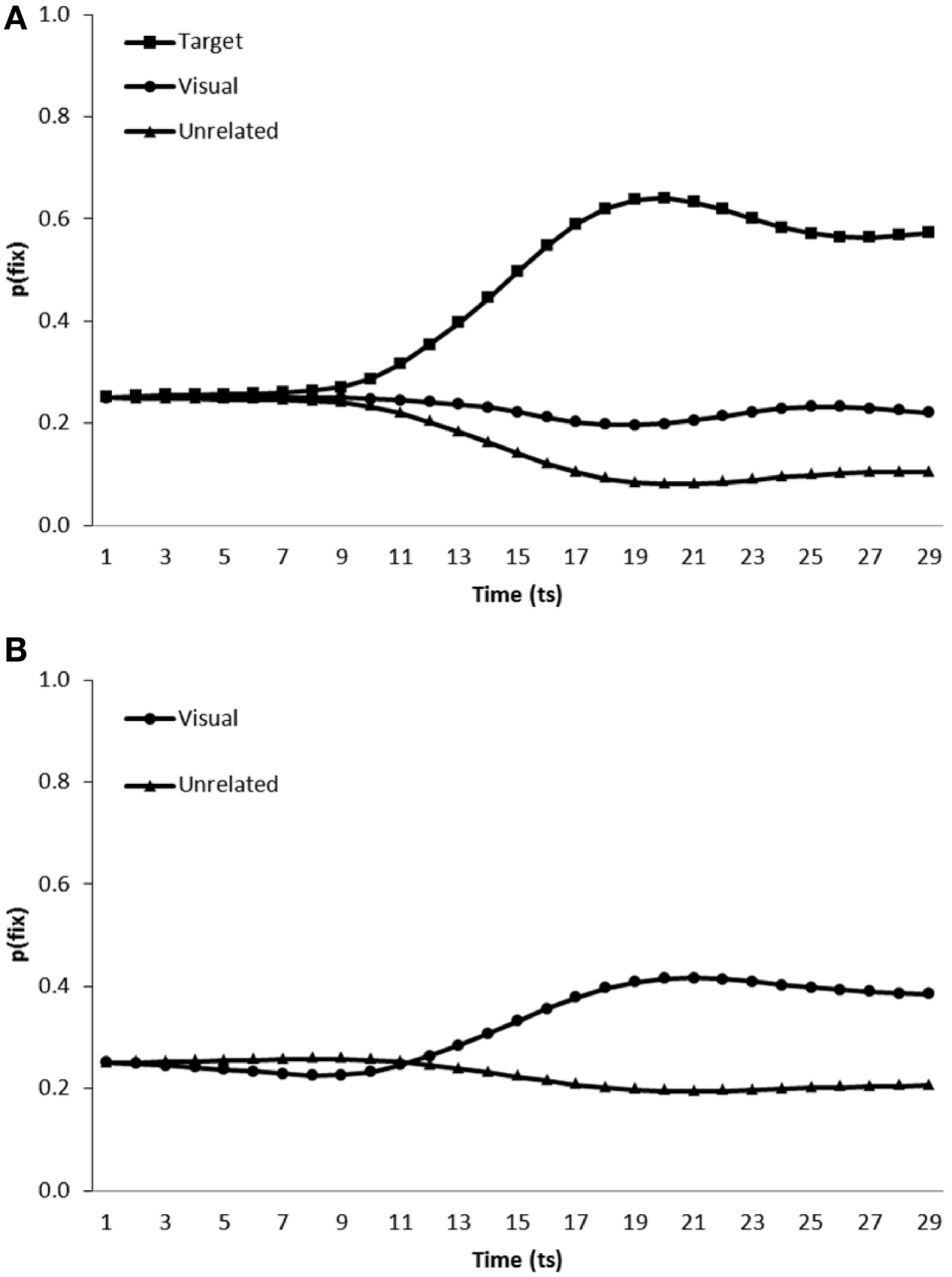
**Proportion of fixations [p(fix)] directed toward items within scenes containing (A) a target, visual competitor and two unrelated distractors (B) a visual competitor and three unrelated distractors**.

***Simulation of visual competitor effects in the VWP.*** To simulate the conditions under which participants were tested in Allopenna et al.'s (1998) study, the model was presented with a visual display containing a target item, a visual competitor and two unrelated distractors. Simulations of Huettig and Altmann (2007) were conducted using a similar approach yet with targets replaced by an additional distractor. In both cases, the visual input representing four items was presented at time step 0. Then onset of the phonology for the target item began at time step 5, to enable pre-processing of the visual information. There were 480 test trials, with each item (*n* = 20) occurring with competitors in all possible spatial configurations (*n* = 24). The model's “gaze” was computed as the Luce ratio of the eye layer units, for the target, competitor, and unrelated distractor item. Figure 3 displays the performance of the model when presented with target present (Figure 3A: simulating Dahan and Tanenhaus, 2005) and target absent (Figure 3B: simulating Huettig and Altmann, 2007) scenes, averaged over each of the six simulation runs of the model. For analysis we calculated the mean fixation proportions [p(fix)] for each category of item (i.e., target, competitor or unrelated distractor) from word onset until the end of the test trial. The ratio was then calculated between the proportion of fixations toward item type A and the sum of the proportion of fixations toward item type A and B. A ratio above 0.5 would indicate greater fixation of item A. Although we would not anticipate substantial variation in model performance across instantiations for completeness this mean ratio (by instantiation and by item) was compared to 0.5 using one sample *t*-tests (cf. Dahan and Tanenhaus, 2005) to test for differences in fixation behavior toward each category of item.

As can be observed from Figure 3, the model fixated target items [mean ratio = 0.75, *t*_1_(5) = 22.42, *p* < 0.001; *t*_2_(19) = 78.50, *p* < 0.001] and visual competitors [mean ratio = 0.60, *t*_1_(5) = 6.91, *p* = 0.001; *t*_2_(19) = 18.18, *p* < 0.001] more than unrelated distractors when scenes contained a target, visual competitor and two unrelated distractors (when by subjects and by items ratios are identical, only one ratio is presented). In target absent scenes, visual competitors were again fixated more than unrelated distractors [mean ratio = 0.58, *t*_1_(5) = 5.37, *p* < 0.01; *t*_2_(19) = 15.290, *p* < 0.001]. The model therefore replicates the increased fixation of visual competitors observed in Dahan and Tanenhaus (2005) and Huettig and Altmann (2007).

**Figure 4:**
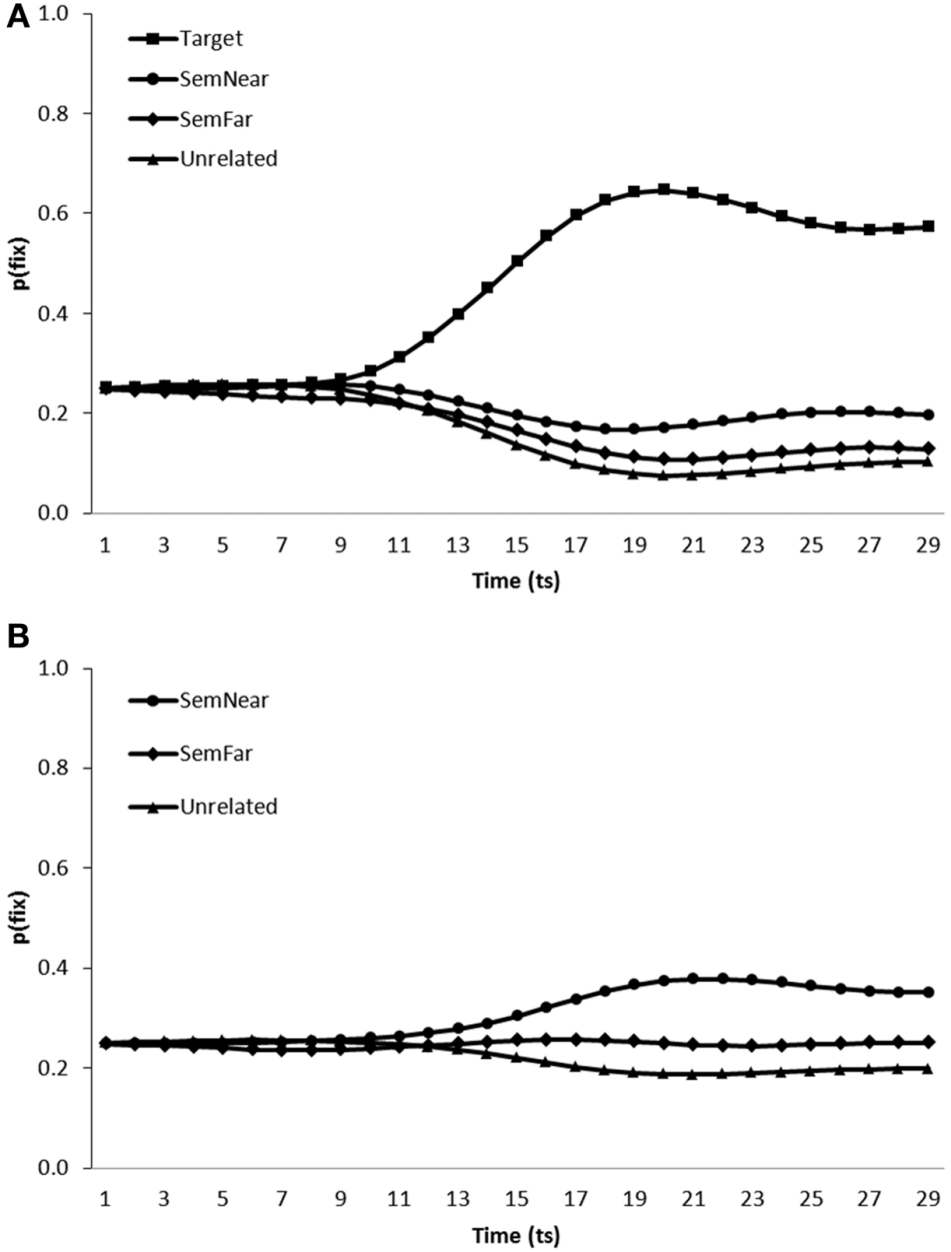
**Proportion of fixations [p(fix)] directed toward items within scenes containing (A) a target, a near semantic neighbor (SemNear), a far semantic neighbor (SemFar) and an unrelated distractor, (B) a near semantic neighbor (SemNear), a far semantic neighbor (SemFar) and two unrelated distractors**.

***Simulation of semantic competitor effects in the VWP.*** We simulated conditions similar to those under which participants were tested in Huettig and Altmann (2005), Yee and Sedivy (2006) and Mirman and Magnuson (2009) by testing model performance when presented with displays containing a near semantic neighbor and a far semantic neighbor in addition to either the target's visual representation and a single unrelated distractor (Figure 4A: simulating Mirman and Magnuson, 2009 and Yee and Sedivy, 2006) or two unrelated distractors (Figure 4B: Simulating Huettig and Altmann, 2005). As for the visual competitor effects, all items were presented in all combinations of positions in the visual input (480 trials in total), and again pre-processing of the visual features of the display were enabled by commencing word onset after a short delay (time step 5). Figure 4 presents the average fixation proportions over time displayed by the model toward each category of item presented in both test conditions.

In target present trials, targets [mean ratio = 0.75, *t*_1_(5) = 25.89, *p* < 0.001; *t*_2_(19) = 79.61, *p* < 0.001], near semantic neighbors [mean ratio = 0.58, *t*_1_(5) = 5.37, *p* < 0.01; mean ratio = 0.57, *t*_2_(19) = 9.89, *p* < 0.001] and far semantic neighbors [mean ratio = 0.52, *t*_1_(5) = 2.82, *p* < 0.05; mean ratio = 0.51, *t*_2_(19) = 4.07, *p* < 0.01] were all fixated more than unrelated distractors. A similar pattern of behavior was observed when the model was tested on target absent trials, with both near [mean ratio = 0.58, *t*_1_(5) = 6.30, *p* < 0.01; mean ratio = 0.57, *t*_2_(19) = 10.67, *p* < 0.001] and far semantic neighbors [mean ratio = 0.53, *t*_1_(5) = 1.80, *p* > 0.1; mean ratio = 0.52, *t*_2_(19) = 7.04, *p* < 0.001] fixated more than unrelated items. Also in-line with behavioral findings far semantic neighbors were fixated less than near semantic neighbors, in both target absent [mean ratio = 0.44, *t*_1_(5) = −3.36, *p* < 0.05; mean ratio = 0.45, *t*_2_(19) = −8.13, *p* < 0.01] and target present [mean ratio = 0.44, *t*_1_(5) = −3.36, *p* < 0.05; mean ratio = 0.44, *t*_2_(19) = −6.97, *p* < 0.001] conditions. The model therefore replicates the increased fixation of semantic competitors in both target absent and target present scenes as observed by Huettig and Altmann (2005) and Yee and Sedivy (2006) respectively, in addition to the graded effect of semantic similarity as reported in Mirman and Magnuson (2009).

**Figure 5:**
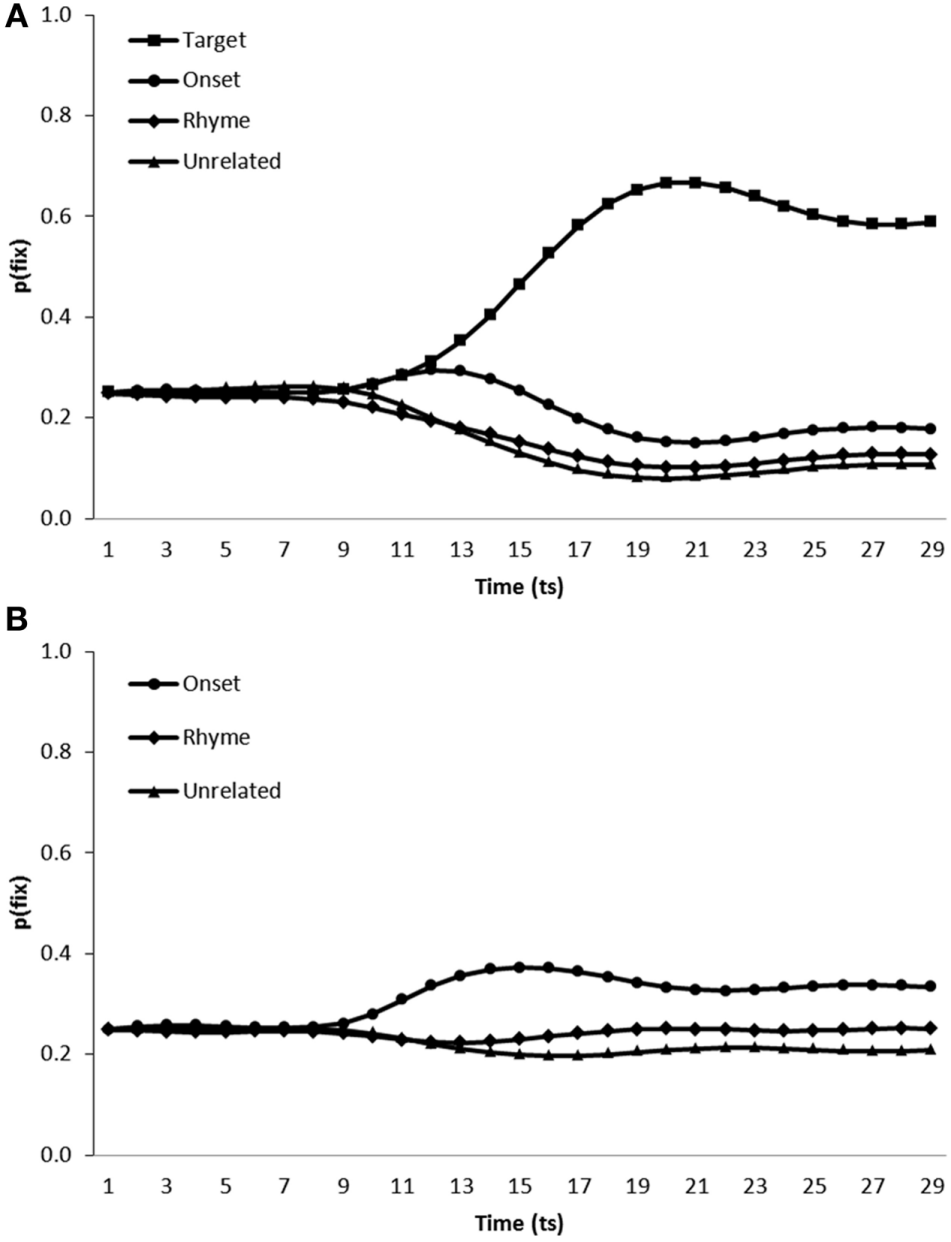
**Proportion of fixations [p(fix)] directed toward items within scenes containing (A) a target, an onset competitor, a rhyme competitor and an unrelated distractor, (B) an onset competitor, a rhyme competitor and two unrelated distractors**.

***Simulation of phonological competitor effects in the VWP.*** To simulate the conditions under which participants were tested in Dahan and Tanenhaus, 2005, the model was presented with scenes containing visual representations of a target item in addition to an onset competitor, a rhyme competitor and an unrelated distractor. For completeness we also tested model performance in a target absent condition (i.e., scenes containing onset competitor, rhyme competitor and two unrelated distractors). In every other way, simulations were conducted exactly as for the visual and semantic competitor simulations. Figure 5 shows the average fixation proportions over time displayed by the model toward each category of item in test displays in both target present (Figure 5A) and target absent (Figure 5B) conditions.

In target present trials, target items [mean ratio = 0.75, *t*_1_(5) = 26.06, *p* < 0.001, *t*_2_(19) = 66.45, *p* < 0.001] and onset competitors [mean ratio = 0.58, *t*_1_(5) = 6.20, *p* < 0.01, *t*_2_(19) = 16.52, *p* < 0.001] were fixated more than unrelated distractors. However, the model fixated rhyme competitors at levels similar to unrelated distractors [mean ratio = 0.51, *t*_1_(5) = 1.75, *p* > 0.1, *t*_2_(19) = 1.69, *p* > 0.1]. On target absent trials both onset [mean ratio = 0.59, *t*_1_(5) = 8.29, *p* < 0.001; *t*_2_(19) = 15.62, *p* < 0.001] and rhyme [mean ratio = 0.53, *t*_1_(5) = 5.62, *p* < 0.01; mean ratio = 0.52, *t*_2_(19) = 3.05, *p* < 0.01] competitors were fixated more than unrelated items. Allopenna et al. (1998) observed increased fixation of both onset and rhyme competitors in target present scenes. Model performance replicated the increased fixation of onset competitors displayed by participants. The model also displayed increased fixation of rhyme competitors although this effect was only clearly observable on target absent trials.

#### Discussion

The model was able to replicate a broad range of single modality word level effects described in the visual world literature, using a single architecture, and incorporating a single shared resource mapping between the modalities. The network replicates findings displaying a bias toward fixating items that overlap with spoken target words in either a visual, semantic or phonological dimension in both target present and absent scenes.

Importantly, the model captures differences in the effect of representational overlap between modalities. The model displays a graded effect of semantic overlap with the probability of fixating semantically related items proportional to the number of semantic features shared between the target and competitor. In a departure from the procedure used in Mirman and Magnuson (2009), within the above simulations both near and far semantic competitors were presented within the same display. Our simulations indicate that far semantic neighbor effects are robust to the additional competition that may result from the presence of closer semantic neighbors within the same scene.

For phonological overlap, the effect was dependent on the temporal location of overlapping features within the representation. Phonological overlap in onsets had a greater influence on fixation behavior than in rhymes, with the latter resulting in only marginal effects of overlap. Although many studies have demonstrated their existence (see Allopenna et al., 1998; Desroches et al., 2006; McQueen and Viebahn, 2007; McQueen and Huettig, 2012), rhyme effects tend to be weak and less robust than onset effects. However, a recent study by McQueen and Huettig (2012) provides evidence that the comparative onset effect is modulated by the level of noise present in the speech signal. They argue that the presence of noise influences the weight placed on initial phonemes as a predictor of the intended word. For example, in a noisy environment sounds heard may not necessarily relate to the identity of the target. Therefore, to make a judgement regarding an item's identity the system benefits from examining evidence from a larger portion of the auditory signal. This work highlights a weakness of current model training and testing, in that the model's learning environment always provided perfect perceptual input of an item in both visual and phonological representations. In the natural learning environment in which participants acquire their knowledge of items, the cognitive system is frequently receiving impoverished representations. This is particularly true in the case of speech, in which factors such as background noise or between speaker variation means that the speech signal received is likely to resemble only a very noisy version of the canonical form. The following simulations extend the model by adding noise to the phonological representations to which the model is exposed during training.

### Modeling language-mediated visual attention in a noisy learning environment

#### Method

To simulate exposure to noisy phonological input in the natural learning environment, the simulations were repeated but with noise applied to the phonological input during the training stage only. Noise was implemented by randomly switching the binary value of each unit within the phonological representation with *p* = 0.2. Noise was randomly generated for each training trial. To ensure comparable levels of performance between fully trained models on all four training tasks, the number of training trials performed was increased by 50%. In all other respects the procedure used to train and test the noisy model was identical to that applied to the previously detailed noiseless model.

#### Results

***Pre-test.*** The noisy model displayed the same high level of performance on both visual to semantic mappings and phonological to semantic mappings as displayed by the noiseless model. In both cases, the noisy model produced activation in the semantic layer most similar (cosine similarity) to the target item's semantic representation for all items within the training corpus.

Performance on orientation tasks was also similar for models trained in both noise conditions. On phonological orienting test trials, the noisy model selected the location of the target on at least 3 of 4 test trials for 99.75% of items in the training corpus. The overall proportion of correct phonological orienting test trials (trials in which the eye unit corresponding to the location of the target was most highly activated) was 87% for the noisy model. When comparing the proportion of correct trials across instantiations between noise conditions, noiseless models performed significantly better than noisy models on this task (*p* = 0.01).

Noisy models correctly selected the location of the target as indicated by the presence of its semantic representation on at least 3 of 4 test trials for all items within the corpus. Overall accuracy on semantic orienting tasks for the noisy model was 90% (σ = 0.02). The difference between noisy and noiseless models was not significant on this task when comparing across instantiations.

**Figure 6:**
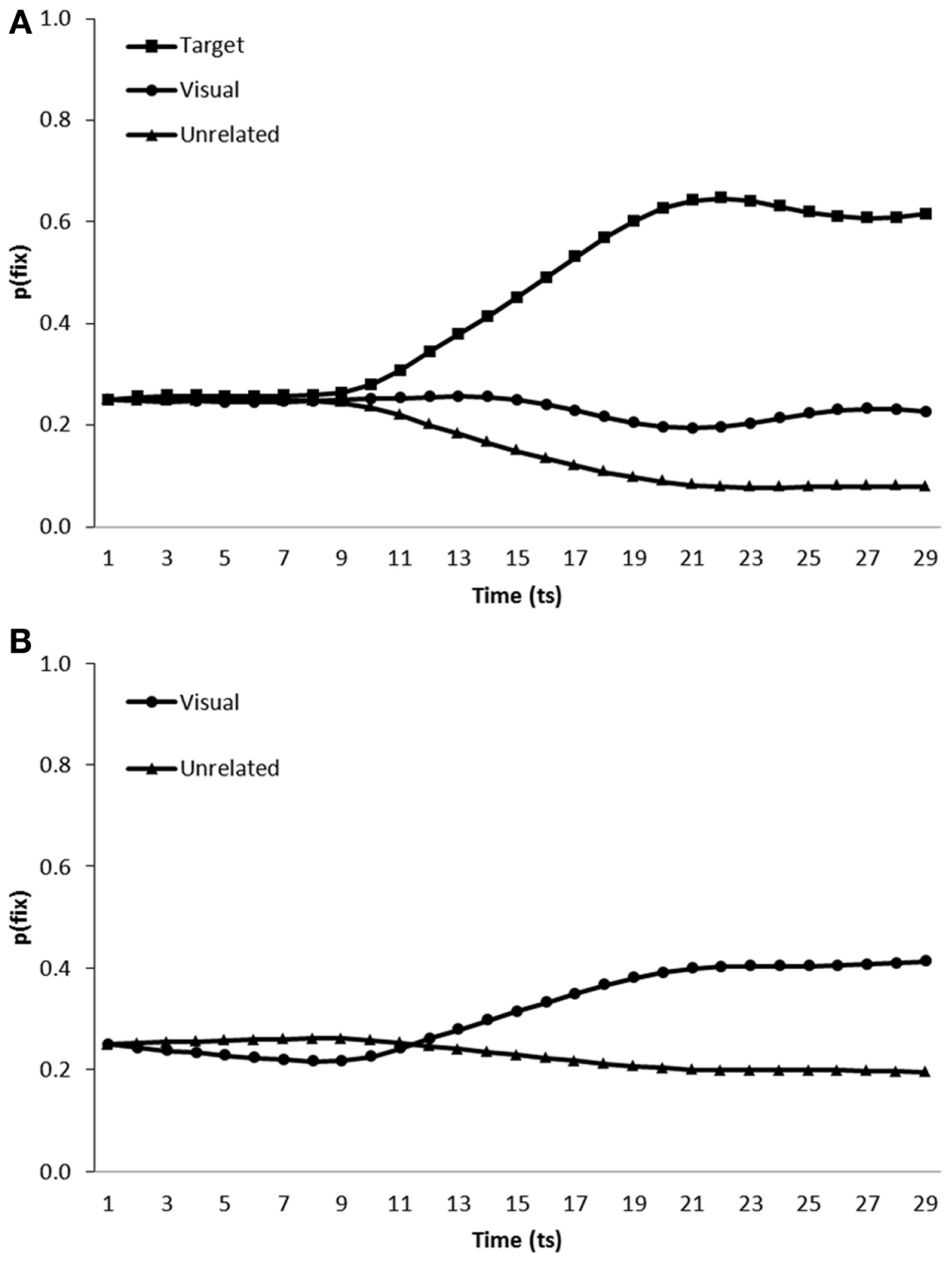
**Proportion of fixations [p(fix)] directed toward items within scenes containing (A) a target, visual competitor and two unrelated distractors (B) a visual competitor and three unrelated distractors; by the model trained in a noisy learning environment**.

***Simulation of visual competitor effects.*** Figure 6 displays the performance of the noisy model when tested on scenes containing a visual competitor in addition to either the visual representation of the target and two unrelated distractors (Figure 6A) or no target and three unrelated distractors (Figure 6B).

On target present trials, both the targets [mean ratio = 0.77, *t*_1_(5) = 27.21, *p* < 0.001; *t*_2_(19) = 89.97, *p* < 0.001] and visual competitors [mean ratio = 0.62, *t*_1_(5) = 7.60, *p* < 0.01; *t*_2_(19) = 22.22, *p* < 0.001] were fixated more than unrelated distractors. Visual competitors were also fixated above distractor levels on target absent trials [mean ratio = 0.60, *t*_1_(5) = 14.52, *p* < 0.001; mean ratio = 0.59, *t*_2_(19) = 18.75, *p* < 0.001].

**Figure 7:**
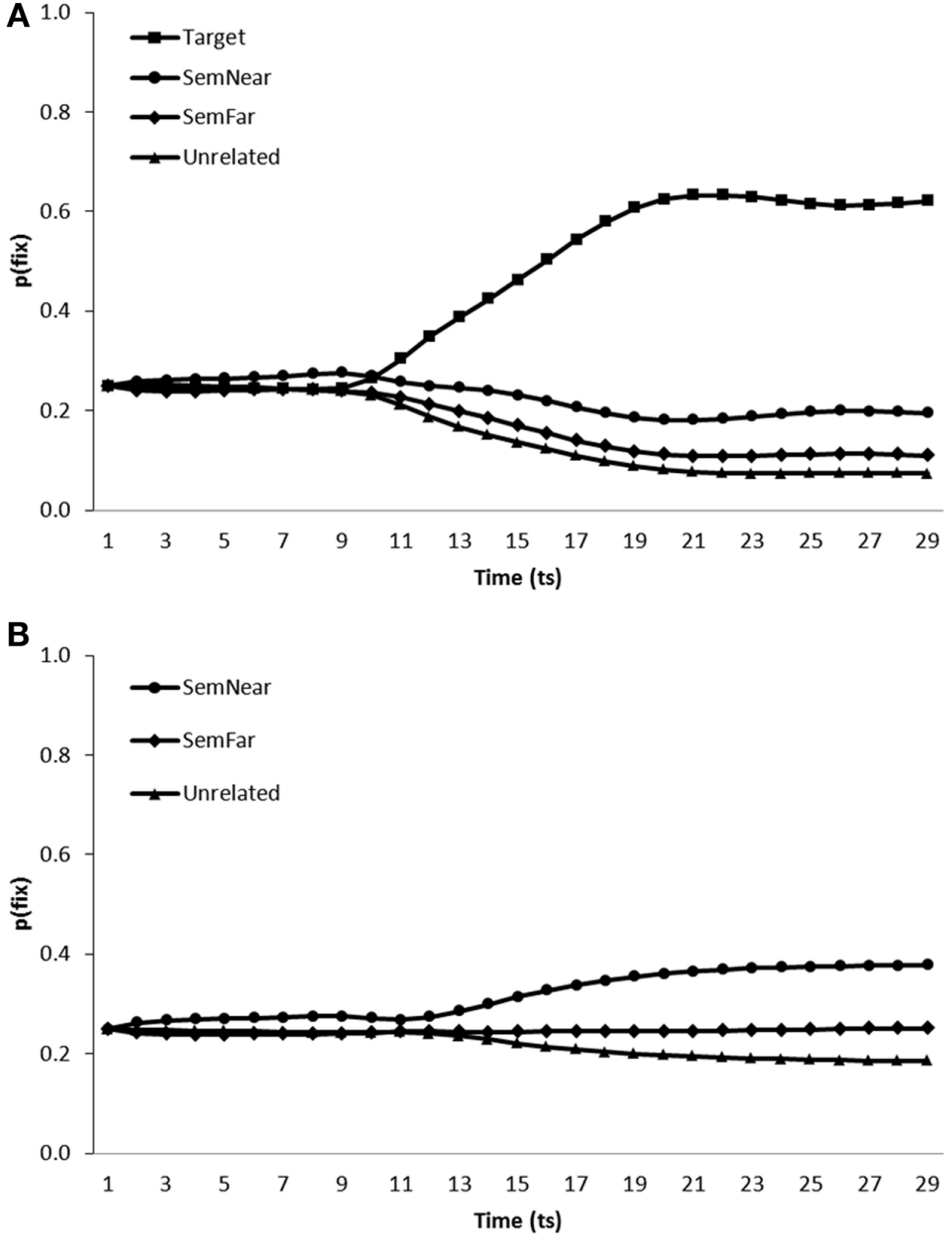
**Proportion of fixations [p(fix)] directed toward items within scenes containing (A) a target, a near semantic neighbor (SemNear), a far semantic neighbor (SemFar) and an unrelated distractor, (B) a near semantic neighbor (SemNear), a far semantic neighbor (SemFar) and two unrelated distractors; by the model trained in a noisy learning environment**.

***Simulation of semantic competitor effects.*** The fixation behavior displayed by the noisy model on trials containing semantic competitors can be seen in Figure 7. The model was tested on scenes containing a near and far semantic neighbor in addition to either the target and a single unrelated distractor (Figure 7A) or no target and two unrelated distractors (Figure 7B).

On target present trials, targets [mean ratio = 0.78, *t*_1_(5) = 29.48, *p* < 0.001; mean ratio = 0.76, *t*_2_(19) = 102.21, *p* < 0.001], near semantic neighbors [mean ratio = 0.62, *t*_1_(5) = 6.42, *p* < 0.01; mean ratio = 0.60, *t*_2_(19) = 18.389, *p* < 0.001] and far semantic neighbors [mean ratio = 0.54, *t*_1 (5)_ = 2.31, *p* < 0.1; mean ratio = 0.52, *t*_2_(19) = 5.934, *p* < 0.001] were all fixated more than unrelated distracters. On target absent trials, both near [mean ratio = 0.60, *t*_1_(5) = 13.78, *p* < 0.001; mean ratio = 0.59, *t*_2_(19) = 22.51, *p* < 0.001] and far [mean ratio = 0.53, *t*_1_(5) = 2.75, *p* < 0.05; mean ratio = 0.52, *t*_2_(19) = 7.13, *p* < 0.001] semantic neighbors were again more likely to be fixated than unrelated items. When comparing between near and far semantic competitors, far neighbors were fixated less than near neighbors both in target present [mean ratio = 0.42, *t*_1_(5) = −12.45, *p* < 0.001; *t*_2_(19) = −12.81, *p* < 0.001] and absent [mean ratio = 0.43, *t*_1_(5) = −11.81, *p* < 0.001; *t*_2_(19) = −15.84, *p* < 0.001] trials.

**Figure 8:**
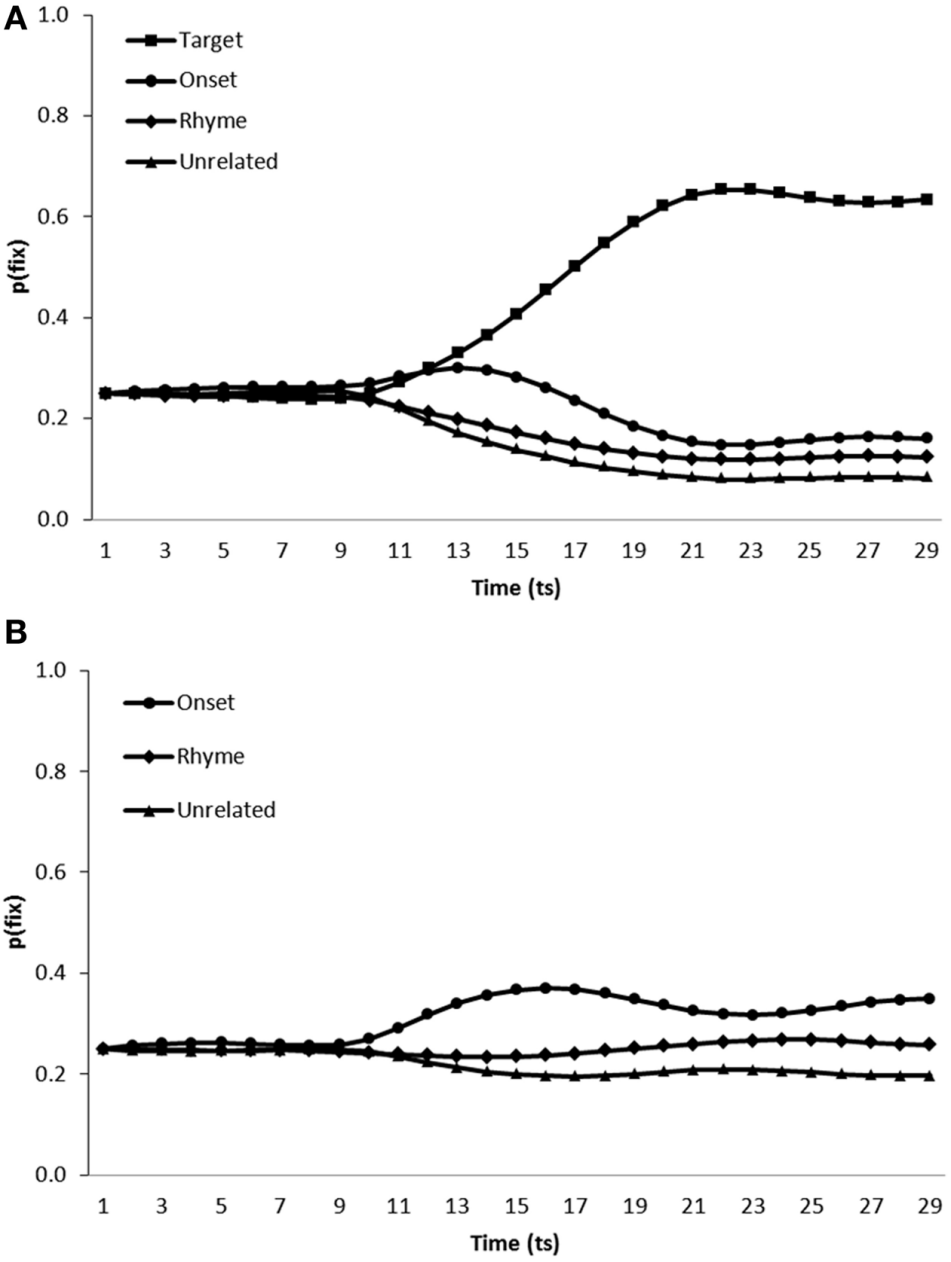
**Proportion of fixations [p(fix)] directed toward items within scenes containing (A) a target, an onset competitor, a rhyme competitor and an unrelated distractor, (B) an onset competitor, a rhyme competitor and two unrelated distractors; by the model trained in a noisy learning environment**.

***Simulation of phonological competitor effects.*** Finally, the model was tested on scenes containing onset and rhyme competitors in addition to either the target and a single unrelated distractor (Figure 8A) or two unrelated distractors (Figure 8B).

In target present scenes, the model displayed increased fixation of target items [mean ratio = 0.77, *t*_1_(5) = 36.71, *p* < 0.001; *t*_2_(19) = 76.149, *p* < 0.001], onset competitors [mean ratio = 0.60, *t*_1_(5) = 6.51, *p* < 0.01; mean ratio = 0.61, *t*_2_(19) = 18.11, *p* < 0.001] and rhyme competitors [mean ratio = 0.54, *t*_1_(5) = 3.13, *p* < 0.05; *t*_2_(19) = 6.842, *p* < 0.001] in comparison to unrelated distractors. Onset [mean ratio = 0.60, *t*_1_(5) = 11.09, *p* < 0.001; *t*_2_(19) = 17.35, *p* < 0.001] and rhyme competitors [mean ratio = 0.54, *t*_1_(5) = 3.13, *p* < 0.05; *t*_2_(19) = 8.90, *p* < 0.001] were also fixated more than distractors in target absent scenes.

#### Discussion

The above results demonstrate that the model of language-mediated visual attention presented in this paper is still able to replicate a broad range of features of language-mediated visual attention when trained in a noisy learning environment. Further, and as predicted, by representing noise in the speech signal during training, we are able to replicate additional features of language-mediated visual attention, specifically sensitivity to rhyme competitors.

## General discussion

The amodal shared resource model presented here offers a description of the information and processes underlying language-mediated visual attention and a potential explanation for how it is acquired. The model accomplishes these effects with minimal imposed constraints on information processing modules or channels, and performance in the model is thus driven by representational structure and the different requirements of forming mappings between the distinct types of information. Language-mediated visual attention is simulated as a function of the integration of past and current exposure to visual, linguistic and semantic forms. The model thereby provides an explicit description of the connection between the modality-specific input from language and vision and the distribution of eye gaze in language-mediated visual attention.

The model replicated the following features of language-mediated visual attention demonstrated in VWP studies: Fixation of onset and rhyme competitors above unrelated distractor levels in target present scenes (Allopenna et al., 1998); (2) Fixation of visual competitors above unrelated distractor levels in target present (Dahan and Tanenhaus, 2005) and target absent (Huettig and Altmann, 2007) scenes; and (3) Fixation of semantic competitors above unrelated distractor levels and relative to semantic relatedness in both target present (Yee and Sedivy, 2006; Mirman and Magnuson, 2009) and absent (Huettig and Altmann, 2005) scenes. A summary of the effects replicated by the model is presented in Table 3.

**Table 3:** **Table comparing the results of both noiseless and noisy simulations with behavioral results reported in the VWP literature**.

**Study**	**Scene**				**Effect A**			**Effect B**		
**References**	**Item 1**	**Item 2**	**Item 3**	**Item 4**	**Behav.**	**Noiseless**	**Noisy**	**Behav.**	**Noiseless**	**Noisy**
Allopenna et al., 1998	Target	**Onset (A)**	**Rhyme (B)**	Dist	√	√ (0.58)	√ (0.60)	√	X(0.51)	√ (0.54)
Dahan and Tanenhaus, 2005	Target	**Visual (A)**	Dist	Dist	√ (0.7)	√ (0.60)	√ (0.62)			
Huettig and Altmann, 2007	**Visual (A)**	Dist	Dist	Dist	√	√ (0.58)	√ (0.60)			
Yee and Sedivy, 2006	Target	**Sem (A)**	Dist	Dist	√	√ (0.58)	√ (0.62)			
Huettig and Altmann, 2005	**Sem (A)**	Dist	Dist	Dist	√	√ (0.58)	√ (0.60)			
Mirman and Magnuson, 2009^*^	Target	**Near Sem (A)**	**Far Sem (B)**	Dist	√	√ (0.58)	√ (0.62)	√	√ (0.52)	(0.54)

The items displayed within scenes in each empirical study are listed with observed competitor effects highlighted in bold. Competitor-Distractor ratios (by subject/instantiation) in parentheses if reported; √, behavioral effect replicated; X, failure to replicate behavioral effect;

* , Study presented near and far semantic competitors on separate trials. Dist, distractor; Sem, semantic competitor; Onset, phonological onset competitor; Rhyme, phonological rhyme competitor.

The results of the above simulations met the objectives of our study as follows. First, the model demonstrates that a H&S model, with minimal computational architectural assumptions, was sufficient for replicating the word level effects of phonological and semantic influences on language processing in the VWP. The simulation results replicate a broad range of the word level effects described within the VWP literature as features of this complex cognitive ability, without requiring separate resources or individually trained pathways between distinct representational information. Second, the model further generalized to replicate the effects of visual similarity in the VWP and sensitivity to the effects of presenting or not presenting the target object in various experimental manipulations of visual, phonological and semantic competitors.

Within our model language-mediated visual attention is described as an emergent property of the structure of representations present in the natural environment and the task demands imposed on the system by that environment. Knowledge of an item is acquired by repeated, simultaneous exposure to its multiple forms. For example, hearing the name of an object while looking at it, or experiencing the function of an item while hearing its name. Such experience leads to associations between the properties defining an object in separate modalities. With repeated and simultaneous exposure to their various forms inhibitory or excitatory connections between such properties are strengthened in order for the system to efficiently map between representations or carry out a given task. In this way, the model provides an explicit and detailed description of how multimodal knowledge of an item is acquired and stored, in addition to how complex multimodal behaviors such as selecting an item based on its function may be achieved and acquired. Thus, the model argues that many word level features of language-mediated visual attention are a necessary consequence of developing multimodal knowledge of items through such a mechanism.

Critically, the model captures contrasts in the effect of overlap in differing modalities. For example, for items that only overlap in a semantic dimension the probability of the model fixating an item is directly proportional to the number of semantic features the two items share. This replicates findings observed in the VWP in which the probability of fixating items has been predicted by semantic norming data (Mirman and Magnuson, 2009) and corpus-based measures of semantic similarity (Huettig et al., 2006). However, in the case of phonological overlap, the temporal location of the overlapping phonemes has a critical influence on the resulting effect. The model replicates the effects of phonological overlap observed in Allopenna et al. (1998) with items that share initial phonemes fixated earlier and with greater probability than items that share phonemes in final positions.

Within the model the level of overlap between target and competitor was strictly controlled both across modalities and between rhyme and onset competitors. Contrasts in fixation behavior toward differing categories of competitor therefore arise as an emergent property of differences in the structural characteristics of representations in each modality. For example, speech unfolds over time. Therefore, phonological representations have a temporal, sequential component not possessed by semantic or visual representations. As the speech signal gradually manifests, early phonemes provide a good, or in the case of a noiseless learning environment they provide a perfect, predictor of the intended word. Therefore, any item that shares the same initial sequence of phonemes with the target is more likely to be fixated by the model. By the time later phonemes are available, the system already has sufficient information, in the case of the noiseless simulations, to identify the target and therefore information provided by later phonemes does not have the opportunity to exert influence on target selection. It is for this reason increased sensitivity to rhyme competitors is displayed by a model trained in a noisy environment compared to one trained in a noiseless environment in which onset phonemes are perfect predictors of the unfolding word. Behavior of the noisy model demonstrates that introducing a low level of noise to speech in the learning environment is sufficient to allow the subtle influence of rhyme overlap to emerge.

This line of argument overlaps with the explanation provided in Magnuson et al. (2003) for the observed reduced sensitivity over the course of word learning to rhyme competitors. They argue that it takes time for the system to learn the value of early phonemes as predictors of the unfolding word. Therefore, at earlier stages of development other overlapping aspects of a word's phonology may exert equal or greater influence on target selection. In a noiseless environment an optimal model should display no influence of rhyme overlap, as sufficient information is carried by initial phonemes to correctly identify the target item. However, in a noisy environment the optimal model would display sensitivity to rhyme overlap proportional to the level of noise in the environment, as this will dictate the probability that the rhyme competitor is the true target given the initially perceived input. Given this line of argument, it is not only external noise that would dictate a system's sensitivity to rhyme overlap but also the level of noise or error within the system itself. For example, noise simulated within the current model could equally reflect errors in phonological perception or fluctuations in attention, the contribution of which could possibly be examined through further combined modeling and VWP studies.

Similar to TRACE (McClelland and Elman, 1986), our model displays sensitivity to overlap in both phonological onsets and rhyme. However, there are differences between the models in their explanation for these effects. As in the model we present, TRACE is able to exploit similarity at all points within the phonological form of the word in terms of co-activating phonological competitors. However, unlike some previous models (Marslen-Wilson, 1987, 1993; Norris, 1994; Magnuson et al., 2003) and the model presented in this paper the disparity between sensitivity to cohort and rhyme competitors in TRACE is not driven by bottom-up mismatch but instead purely by onset competitors accumulating activation prior to rhyme competitors due to their inherent temporal advantage (Magnuson et al., 2003).

Many similarities are shared between our computational model and the theoretical model of language-mediated visual attention proposed in Huettig and McQueen (2007). Both models argue that behavior in the VWP is driven by matches between information extracted from visual and auditory input at phonological, semantic and visual processing levels. However, they differ subtly in how this is implemented. Huettig and McQueen suggest that contrasts in fixation dynamics displayed toward each category of competitor are driven by aspects of the systems architecture, specifically temporal contrasts in the nature of the cascade of information between modalities. For example, they argue that early fixation of phonological competitors reflects earlier activation of phonological representations in the speech-recognition system, with activation then later cascading to semantic and visual levels of processing, which in turn leads to the later increased fixation of visual and semantic competitors. In contrast, in the model proposed in the current paper, eye gaze is a continuous measure of the simultaneous integration of information activated across all three modalities. Therefore, activation of an item's phonological representation cannot influence gaze independent of currently activated visual and semantic representations.

Huettig and McQueen (2007) highlight the value of the VWP as a tool for probing finer aspects of the architecture of the cognitive system, as eye gaze offers a fine grained measure of the information activated over time. By combining this rich behavioral measure with the current model it may be possible to further examine more subtle aspects of the systems architecture that have so far proved difficult to isolate without implementation. We hope to test whether the parsimonious architecture presented in this paper is compatible with the data provided by Huettig and McQueen (2007). It remains to be seen whether such an architecture can also offer explanation for the complex time course dynamics that emerge when competitors from multiple modalities are presented simultaneously within the same display. The results of our simulations establish the applicability of the shared resource model to account for interactions between pairs of modalities. We demonstrate its ability to replicate a range of effects involving visual-semantic and visual-phonological interactions (see Table 3), a necessary precursor before extending to multiple interactive effects.

Within the model we present, noise is only applied to phonological input. However, in the human cognitive system, perceptual input from all modalities provides only a noisy representation of the true nature of objects in the environment. It may therefore be interesting to also extend the model to capture environmental noise in visual input. Unlike speech, visual descriptions of objects can often be improved by gathering additional information regarding its visual features over time. The literature indicates that certain groups of visual features are activated earlier than others, for example low spatial frequency information has been shown to be recruited early and rapidly by the visual system (Bar, 2003). A detailed implementation of such features of visual processing is yet to be implemented within the model. It is possible that such features may have interesting consequences for language-mediated visual attention. The model described in this paper potentially provides a means of exploring such questions.

Further applications of the model can be found in on-going experimental work that suggests that the relative influence of representational overlap in semantic, visual and phonological dimensions fluctuates over the course of child development (Mani and Huettig, in preparation). As previously discussed, model training simulates the interactions between the cognitive system and the learning environment through which the system acquires knowledge of objects in the world. Through sampling performance of the model as it moves through the training process it is possible to extract measures of its behavior on individual tasks across the course of development. It may therefore be possible, in this way, to explore the developmental story of language-mediated visual attention and provide an explicit description of the mechanism driving observed variation across development.

The model also provides scope for modeling individual differences in language-mediated visual attention observed between mature populations. In a recent study conducted by Huettig et al. (2011c), language-mediated visual attention varied as a consequence of literacy training. Their results showed that whereas a high-literate population demonstrated phonological competitor effects similar to those previously discovered (Allopenna et al., 1998), low-literates' eye gaze did not display sensitivity to phonological overlap between spoken target words and items presented in a visual display. Instead low-literates' gaze was strongly influenced by semantic relationships between items. One explanation for this difference that could be tested in the current model is whether observed differences in language-mediated visual attention between low and high literates emerge a consequence of finer grained processing of the speech signal that follows from increased literacy training (cf. Ziegler and Goswami, 2005). The modeling framework presented in this paper allows manipulation of environmental variables such as the form of representations processed and the tasks performed in the learning environment. By manipulating such variables, it becomes possible to test theoretical explanations for these observed individual differences (see Smith et al., 2013).

As in previous H&S models, emergent properties of this style of model are dictated by multiple factors including environmental variables such as the structure of representations and the type and frequency of mappings performed, in addition to resource-related factors such as the number of units within the central resource. With so many degrees of freedom open to the modeler with which to fit H&S models to data sets, it is crucial that steps are taken to avoid simply data fitting and instead develop a model able to probe important theoretical questions (see Seidenberg and Plaut, 2006). Any assumptions made in the model development process should be justifiable with clear theoretical motivation. One effective method of model validation is to extract from a model testable non-trivial predictions. Our model of VWP effects was effective in simulating a broad range of behavior using a single set of parameters. When noise was present in the training environment, we effectively simulated processing of visual, phonological and semantic competitors and in differing situations—when targets were present or absent from the visual input to the model. Furthermore, subtle patterns of fixations over time were demonstrated by the model that were similar to behavioral data. Figure 1 illustrated the effect of semantic competitors in behavioral data, with an emerging preference for the target, and a later, but smaller, diverging effect of near and distant semantic competitors. A similar pattern is illustrated in the model, as shown in Figure 4. Data-fitting to such nuanced patterns of behavior is likely to require many free parameters, and so our model's dynamics are effective in generalizing to a broad range of behavioral effects.

Connecting modalities via a central resource as in H&S does not provide the only solution for connecting the various modalities known to play a role in language-mediated visual attention. Other models are possible in which one builds in additional modalities separately. The advantage of the model presented in this paper is that the effects reported are emergent. A critical feature of the model's architecture is the amodal shared resource that intervenes between modal-specific representational systems. Such an architecture is characteristic of H&S models (Plaut, 2002; Rogers et al., 2004; Dilkina et al., 2010), and ensures that no unnecessary assumptions about how mappings are formed between distinct representations are included in the model. Furthermore, the amodal shared resource in the model appears to parsimoniously support the interactions between multiple representations that are so characteristic of complex language-processing behavior. Processing in each of the modalities described within the model is likely to involve complex hierarchical systems (see Simmons and Barsalou, 2003; McNorgan et al., 2011). However, the results of our study demonstrate that a parsimonious H&S architecture is able to capture a diverse range of effects reported in the language-mediated visual attention literature (see Table 3). We argue that the model presented operates at a suitable level of abstraction to act as a meaningful proxy for the cognitive system that supports language-mediated visual attention. In doing so the model provides a valuable contribution in describing the nature of the representations and processes involved in this complex multimodal behavior performed by individuals on a daily basis, and further it offers a tool through which the factors driving individual differences in language-mediated visual attention can be examined.

### Conflict of interest statement

The authors declare that the research was conducted in the absence of any commercial or financial relationships that could be construed as a potential conflict of interest.
